# Believing and sharing misinformation, fact-checks, and accurate information on social media: The role of anxiety during COVID-19

**DOI:** 10.1177/14614448211011451

**Published:** 2023-01

**Authors:** Isabelle Freiling, Nicole M Krause, Dietram A Scheufele, Dominique Brossard

**Affiliations:** University of Vienna, Austria; University of Wisconsin—Madison, USA; University of Wisconsin—Madison, USA; Morgridge Institute for Research, USA

**Keywords:** Anxiety, COVID-19, fact-check, misinformation, partisan motivated reasoning, corrections, social media

## Abstract

The COVID-19 pandemic went hand in hand with what some have called a “(mis)infodemic” about the virus on social media. Drawing on partisan motivated reasoning and partisan selective sharing, this study examines the influence of political viewpoints, anxiety, and the interactions of the two on believing and willingness to share false, corrective, and accurate claims about COVID-19 on social media. A large-scale 2 (emotion: anxiety vs relaxation) × 2 (slant of news outlet: MSNBC vs Fox News) experimental design with 719 US participants shows that anxiety is a driving factor in belief in and willingness to share claims of any type. Especially for Republicans, a state of heightened anxiety leads them to believe and share more claims. Our findings expand research on partisan motivated reasoning and selective sharing in online settings, and enhance the understanding of how anxiety shapes individuals’ processing of risk-related claims in issue contexts with high uncertainty.

The emergence of the COVID-19 pandemic has been accompanied by what the World Health Organization (WHO) has called an “infodemic,” or a crisis of misinformation about the SARS-CoV-2 virus and strategies to mitigate its spread (Krause et al., 2020). While there is limited evidence suggesting a more rapid spread of misinformation for COVID-19 as compared to other scientific or political issues, some misleading claims gained visibility because they were endorsed by political leaders, including former President Trump of the United States. In April and May of 2020, based on evidence in the medical journal *The Lancet*, major media outlets and fact-checkers attempted to clarify (or even debunk) the former President’s claims that hydroxychloroquine was a promising cure for COVID-19. In early June, the scientific studies forming the basis of these fact-checks were retracted (Rabin and Gabler, 2020). Right-wing media outlets such as *Breitbart* quickly asserted that the retractions behind so-called “Lancetgate” were “a humiliation” for the political left, broadly painting efforts to establish or verify “facts” about COVID-19 as a political war in which scientists’ and fact-checkers’ statements have “more to do with political activism than with disinterested science” (Delingpole, 2020).

This example illustrates two challenges for fact-based communication during pandemics like COVID-19. First, informational interventions amid rapidly changing health crises are complicated by the fluid nature of scientific knowledge. Corrections that are based on science that might turn out to be wrong or in need of revision can undermine long-term trust in science (Scheufele et al., 2020). Second, the COVID-19 misinfodemic is a multi-layered issue in which, at least in the United States, politically polarized views of the virus are intertwined with similarly divergent perceptions of “fake news” (Krause et al., 2020).

Although over one-third (35%) of the US population reports using Facebook to obtain news (Newman et al., 2020), there is limited work on how views about risks like COVID-19 *interact* with views of misinformation to affect encounters with claims on social media. Addressing this problem empirically, this article experimentally investigates the role of partisanship, extremity of ideology, ideological source congruency, and anxiety in individuals’ expressed belief in and willingness to share Facebook posts containing misinformation on COVID-19, with fact-checked and accurate claims for comparison. “[M]isinformation concerning health has particularly severe consequences with regard to people’s quality of life and even their risk of mortality” (Swire-Thompson and Lazer, 2020: 434)—indeed, there are already cases of unsubstantiated claims about COVID-19 leading to death (Shepherd, 2020). Despite the previously mentioned complexities of defining “the facts” about a rapidly changing crisis, there is a clear need to better understand how people process claims about highly impactful risks beset by tremendous scientific uncertainty. Because COVID-19 was a novel and emerging risk at the time of this study, and because the information environment around it was similarly shaped by uncertainty, anxiety is a relevant emotion in this issue context (Holman et al., 2020). We, therefore, focus specifically on anxiety rather than other emotions that are also known to influence belief in (mis)information, such as anger (Weeks, 2015). COVID-19 is not the first issue of its kind, and it certainly will not be the last.

## Misinformation in online media: an old adversary in a new light

Misinformation is a multidimensional concept (Scheufele and Krause, 2019). Here, we define “misinformation” as claims—well-intentioned or not—that are at odds with the best available empirical evidence. We adopt this definition for two reasons.

First, defining misinformation relative to its temporal context is essential for rapidly evolving pandemics like COVID-19 where what counts as the “best available evidence” is constantly shifting. Our example of hydroxychloroquine shows that scientific “facts” established early in a crisis are likely the result of fast peer review and limited replication, raising the chance they will be corrected or at least modified by subsequent research. What is true about COVID-19 today may turn out to be false tomorrow (Marcus and Oransky, 2020).

Second, although there is clearly a difference between “disinformation” that is purposely false and “misinformation” that may be well-intentioned despite its falsity, our definition is agnostic to intent. Even in the absence of malintent, audiences may still perceive claims to be malicious, and perception may matter more than reality when looking at individual-level processing of claims about a deadly risk.

Misinformation is not new, nor unique to social media. False claims have long existed in legacy media, but new media present novel or enhanced concerns about falsehoods, raising the stakes of our efforts to combat misinformation online (Katz and Mays, 2019; Krause et al., 2019). Specifically, algorithmic curation of content can bias information diets for consumers (Hargittai et al., 2020). (Mis)information congruent with individuals’ predispositions—such as political beliefs—is likely to be algorithmically prioritized on social media timelines in order to maximize user engagement and retention (Cacciatore et al., 2016; Standage, 2020). Furthermore, emotionally evocative information sparks more user-driven diffusion online (Brady et al., 2017), including for scientific topics (Milkman and Berger, 2014), so new media users may be more likely to see (mis)information that resonates emotionally, facilitating affective and expressive responses (Haidt and Rose-Stockwell, 2019; Papacharissi, 2015).

While there are arguably many ways to address the broad topic of interest to us here—that is, factors shaping individuals’ reactions to science-related misinformation online—the earlier evidence highlights the value of focusing on individuals’ strongest and most relevant cognitive predispositions, as well as emotions. In the context of COVID-19, we thus investigate the interplay of partisan information processing, partisan selective sharing, and anxiety in shaping individuals’ encounters with (false) claims on Facebook.

## (Mis)information processing: one partisan’s trash is another partisan’s treasure

In the United States, partisanship functions as a powerful “heuristic” (Fiske and Taylor, 1991; Popkin, 1994), influencing how people form beliefs about political and scientific topics, including risks to public health (see, for example, Achen and Bartels, 2017; Cacciatore et al., 2013; Chinn and Pasek, 2020; Druckman and Bolsen, 2011; Kraft et al., 2015; Yeo et al., 2014). With COVID-19, we are already seeing polarized attitudes emerging along partisan lines (Scheufele et al., 2020).

### Partisan information processing

When exposed to information that conflicts with their previously held beliefs, individuals can experience “cognitive dissonance” (Festinger, 1957), a state of psychological unease that may activate “motivated reasoning” (Kunda, 1990), or biased information processing in which people defend their prior beliefs and identities. “Partisan motivated reasoning” specifically defends political identities (Leeper and Slothuus, 2014) and can occur for issues at the intersection of politics, science, and risk. For example, individuals at opposite ends of the political spectrum who see the same information about climate change form polarized perceptions favoring their prior beliefs (e.g. Hart and Nisbet, 2012), sometimes including belief in misinformation (Nyhan and Reifler, 2010).

However, another explanation for these same outcomes is that individuals simply possess different beliefs about *which sources* are accurate (Druckman and McGrath, 2019), since source credibility and trustworthiness are known to influence beliefs (Metzger et al., 2010; Pornpitakpan, 2004; Wilson and Sherrell, 1993). Notably, the effect of source cues in news contexts (Freiling, 2019; Tandoc, 2019) is evident in US polls. For 48% of Americans, the news outlet has a large impact on a story’s perceived credibility (Barthel and Mitchell, 2017). Work with ideologically slanted news sources specifically shows that individuals perceive attitude-consistent sources as more credible than attitude-inconsistent sources (Metzger et al., 2020).

Given that individuals’ political views can act as heuristics, we expect the following:

*H1a.* Individuals will believe more in claims from an ideologically congruent source than in claims from an ideologically incongruent source.

It is important to note that our use of the term “claims” here includes false, accurate, and fact-checked claims. If we mean to make predictions about only some kinds of claims, we will specify this in subsequent hypotheses or research questions.

### Partisan selective sharing

Beyond influencing attitudes and beliefs, partisan information processing also influences behaviors, such as sharing (mis)information. This is particularly relevant in new media environments, where individuals can recirculate information with ease.

On social media, individuals are more likely to trust an article shared by a trusted public figure, increasing chances of engagement (Sterrett et al., 2019). Individuals are also more likely to recirculate messages (especially fact-checks) that align with their politics (Shin and Thorson, 2017), and the visibility of sharing may encourage people to pay close attention to their identities when doing so (Shin and Thorson, 2017). Thus, we expect the following:

*H1b.* Individuals will be more willing to share claims on social media published by an ideologically congruent source than claims published by an ideologically incongruent source.

### Extremity of ideology

In some cases, people with greater commitment to their political views are the ones most likely to engage in motivated reasoning (see, for example, Lodge and Taber, 2013; Miller et al., 2016). One explanation for this is that people with well-developed ideologies can more effectively identify how and if new information fits existing mental schema (Converse, 1964). Furthermore, when strong partisans hold misperceptions, they have been shown to exhibit greater confidence that their views are correct (Kuklinski et al., 2000), and individuals with more extreme ideologies tend to “dig in” to their beliefs when they are exposed to correctives (Nyhan and Reifler, 2010). However, this “backfire effect” has been challenged by unsuccessful attempts to fully replicate it (Wood and Porter, 2018). Still, we expect the following:

*H2a.* The more extreme individuals’ political ideology is, the more they will believe claims published by an ideologically congruent source.
*H2b.* The more extreme individuals’ political ideology is, the more they will be willing to share claims published by an ideologically congruent source.

### Partisan information processing and sharing in the context of COVID-19

Some work has asked whether conservatives or liberals are systematically more likely to engage in biased information processing, and there is some evidence that right-leaning individuals exhibit greater belief in misinformation due to both motivated reasoning (Nyhan and Reifler, 2010) and selective exposure (Kull et al., 2003). However, these findings may be attributable more to the specific issues discussed in these studies than to innate differences among these groups.

Therefore, when the politicization of an issue aligns falsehoods with one side of politics, we would expect to see more misperceptions among that partisan group. In the context of COVID-19, the purveyance of misleading claims by former President Trump (a Republican) to downplay the virus (Calvillo et al., 2020) offers reason to expect the following:

*H3a.* Republicans will be more likely to believe misinformation about COVID-19 than Democrats.
*H3b.* Republicans will be less likely to believe accurate information about COVID-19 than Democrats.

Furthermore, we expect partisan-driven effects to be enhanced by ideological extremity:

*H4a.* Extremity of ideology will moderate the effect of partisanship on belief in misinformation about COVID-19, in that the difference between Republicans’ and Democrats’ belief in misinformation will be greater when extremity of ideology is higher.
*H4b.* Extremity of ideology will moderate the effect of partisanship on belief in accurate information about COVID-19, in that the difference between Republicans’ and Democrats’ belief in accurate information will be greater when extremity of ideology is higher.

Finally, given the issue context and research from the United Kingdom showing that conservatives are more likely to share misinformation (Chadwick and Vaccari, 2019), we expect the following:

*H5.* Republicans will be more willing to share misinformation about COVID-19 than Democrats.

As we have argued, individuals’ use of partisanship as a heuristic is likely to influence whether they believe and share false claims about COVID-19 or other topics. However, new media can also encourage information processing that is colored by emotions. In the context of COVID-19, one such emotion is likely to be anxiety.

## Anxiety in encounters with misinformation: a double-edged sword

Although previous studies investigating links between affect and misperception have explored a variety of emotions (e.g. Weeks, 2015), this study focuses more specifically on anxiety. Especially during the early stages of the pandemic, uncertainties about the spread of the virus, the success of likely therapies, and the timing of vaccine interventions all contributed to a climate of increased anxiety (American Psychiatric Association, 2020). This is not overly surprising, given that anxiety is, by definition, an emotion that arises when individuals experience uncertainty about negative outcomes—that is, when they experience “risks,” or the possibility that an undesirable state of reality may occur (Albertson and Gadarian, 2015; Renn, 1992). Furthermore, uncertainties about the science related to COVID-19 and public health responses were exacerbated by the risks of an emerging climate of misinformation (Krause et al., 2020), contributing to public confusion about what was true and false, possibly compounding already-existing anxieties about the virus (Wiederhold, 2020).

Interestingly, the effect of anxiety on information processing and belief formation may not be entirely straightforward. Although anxiety can foster openness to information (Albertson and Gadarian, 2015; Redlawsk et al., 2010; Weeks, 2015)—perhaps, mitigating political bias—it can also enhance susceptibility to attitude-inconsistent information, even if the claims are false (Weeks, 2015). People who “experience lack of control,” as can be the case when being in a general state of high anxiety, may “compensate with strategies that lead to greater acceptance of misperceptions” (Nyhan and Reifler, 2012: 17). We, therefore, expect the following:

*H6.* The more anxious individuals feel, the more they will believe claims about COVID-19.

The effects of anxiety on individuals’ willingness to *share* information are less clear. While some literature links anxiety to increased sharing (Berger, 2011), especially in political contexts with low knowledge (Heiss, 2020), other work shows the opposite (Lerner and Keltner, 2001), with the rationale that “anxiety may cause people to withdraw and avoid risk” (Hasell and Weeks, 2016: 646). Thus, we ask the following:

*RQ1.* How does anxiety influence the willingness to share claims?

## When anxiety and political views collide

Literature on the political psychology of anxiety offers mixed evidence about whether anxiety reinforces or mitigates partisan biases (for an overview, see Albertson and Gadarian, 2015), suggesting the need for more clarity as to how the two constructs can interact in different issue contexts. Research tracing liberals’ and conservatives’ moral psychology suggests there should be differences in how they respond to threats. However, the role of moral tendencies relative to partisan motivated reasoning may depend on the issue context. Although conservatives are generally more susceptible to threats, “including the threat of germs and contamination” (Haidt, 2012: 279), they, in fact, perceive *less* threat from COVID-19 (Calvillo et al., 2020).

Given this discrepancy and the high degree of politicization around COVID-19, it seems that partisan motivated reasoning may be overwhelming moral intuitions (Klein, 2020). Republicans might be pushed not only to downplay the virus, consistent with partisan leadership, but also to avoid “overreacting.” For Democrats, partisan motivated reasoning would theoretically work the opposite way for COVID-19. They would be motivated to maintain their anxiety and possibly perform it for others, showing that they are reacting to COVID-19 with an “appropriate” level of concern. Given these mixed signals, we ask the following:

*RQ2.* How do political views and anxiety interact to influence individuals’ (a) belief in and (b) willingness to share claims?

## Method

### Study design and participants

This study used a 2 (emotion: anxiety vs relaxation) × 2 (news outlet: MSNBC vs Fox News) experimental design with random assignment, embedded in an online survey. We recruited through Amazon Mechanical Turk (MTurk) from late April to early May 2020. Although MTurk samples differ from nationally representative samples, recent work has found that they do not lead to false negatives and positives, nor to inaccurate effect sizes, and that they are more representative than in-person convenience samples (Berinsky et al., 2012; Mullinix et al., 2016).

A total of 719 responses were analyzed after removing 48 participants that did not follow instructions for the writing exercise related to the emotion assigned to them. Of course, those 48 participants likely differ from those who followed instructions. We, nonetheless, chose to exclude them given that we could not verify that they were in the respective emotional state they should have been based on our manipulation. Participants’ ages ranged from 18 to 76 (*M* *=* 39.39, *SD* *=* 11.91). The sample consists of 58.3% males, 40.6% females, 0.3% genderfluid or non-binary individuals, with 0.8% preferring not to answer. The sample was highly educated with only 1 in 10 (10.7%) having a high school diploma, a quarter of respondents (24.3%) having some college education, half of the respondents (49%) having a college diploma, and 14.9% having a graduate school degree or more.

### Procedure

Prior to stimuli exposure, we asked participants a battery of questions, including their political ideology and partisanship. To manipulate emotion, we asked participants to write for 2 minutes about a situation that made them extremely anxious or relaxed (depending on condition assignment) and about the emotion they felt. After a manipulation check, we showed, in a random order, six Facebook posts that we made to look like news article posts about COVID-19, shared either by Fox News or MSNBC (depending on condition assignment). To provide an array of information, we presented two posts containing misinformation, two containing corrective information, and two containing accurate information. The corrective information took the form of a fact-check (see Figure 2 in Appendix).

To keep claims as comparable as possible across conditions, we chose two claims each for misinformation, fact-checks, and accurate information, with one of them being clearly political and the other one being as apolitical as possible. Since each stimulus was shown to every participant, the specific topics could not be identical across the different kinds of claims. Specifically, we chose topics for each claim that were actually circulating at the time (e.g. we chose real-world misinformation for the misinformation claims, real-world fact-checks for the fact-checks, etc.). For each stimulus, participants reported belief in the claim and willingness to share the post. After the experiment, participants reported demographics.

### Measures

*Belief in statements* reflected perceptions of a claim’s accuracy. We asked, “To the best of your knowledge, is the claim in the above post true or false?” (1 = definitely false, 2 = likely false, 3 = likely true, 4 = definitely true, 5 = don’t know). We recoded “don’t know” as the middle category (3) (belief in misinformation: *M* = 2.43, *SD* = 1.27; fact-checks: *M* = 3.49, *SD* = .90; accurate information: *M* = 3.46, *SD* = 1.07).

*Willingness to share* was asked on a 7-point scale (1 = extremely unlikely, 7 = extremely likely): “If you were to see the above on social media, how likely would you be to share it?” (willingness to share misinformation: *M* = 2.54, *SD* = 1.80; fact-checks: *M* = 2.77, *SD* = 1.77; accurate information: *M* = 2.95, *SD* = 1.81).

*Partisanship* was measured on a 7-point scale (1 = strong Democrat, 7 = strong Republican, 4 = Independent): “Generally speaking, do you usually think of yourself as a Republican, Democrat, Independent, or what?” (*M* = 3.68, *SD* = 1.98).

*Extremity of ideology* was measured using two items on a 7-point scale (1 = very liberal, 7 = very conservative, 4 = moderate): “The terms ‘liberal’ and ‘conservative’ may mean different things to people, depending on the kind of issue one is considering. Many people’s views do not fit perfectly into one of the categories below, so please indicate which one you think could best align with your views. (a) economic issues, and (b) social issues.” We merged these two items into an ideology index (1–7) and then coded the ideological extreme values high and the ideological moderate values low.^1^

*Ideological source congruency* was coded based on participants’ ideology and the news outlet condition assignment (Fox News vs MSNBC). We chose ideology over partisanship to measure ideological source congruency because by measuring ideology on social and economic issues we have a more specific measure of participants’ ideological stance that might better reflect their ideological source congruency in combination with the source. Conservatives in the Fox News condition were coded 1 for source congruency, as were liberals in the MSNBC condition. Scores of 4 on the ideology index were coded 0; and participants in an ideologically incongruent condition (i.e. conservatives seeing MSNBC and liberals seeing Fox News) were coded –1.

*Anxiety* was measured, along with other emotions meant to conceal the study’s focus on anxiety, using three items (Weeks, 2015) on a 7-point scale (1 = not at all, 7 = extremely): “Below are some adjectives that describe about how people may feel. Using the scale provided, please indicate how well each adjective describes how you currently feel. (a) anxious, (b) afraid, (c) nervous.” We merged them into an index (1–7) (*M* = 2.99, *SD* = 1.86, Cronbach’s α = .92).

*Manipulation check*. Among the list of items representing other emotions were also the following for relaxation: (a) relaxed, (b) peaceful, and (c) calm. We merged them into an index (1–7) (*M* = 4.60, *SD* = 1.74, Cronbach’s α = .93). Independent sample T-tests showed that our manipulation worked. Individuals in the anxiety condition were significantly more anxious (*M* = 3.46, *SD* = 1.84) than those in the relaxation condition (*M* = 2.55, *SD* = 1.77), *t*(717) = 6.77, *p* < .001. Also, individuals in the anxiety condition were significantly less relaxed (*M* = 4.08, *SD* = 1.85) than those in the relaxation condition (*M* = 5.08, *SD* = 1.48), *t*(665.77) = 8.01, *p* < .001.

## Results

**Figure 1.**
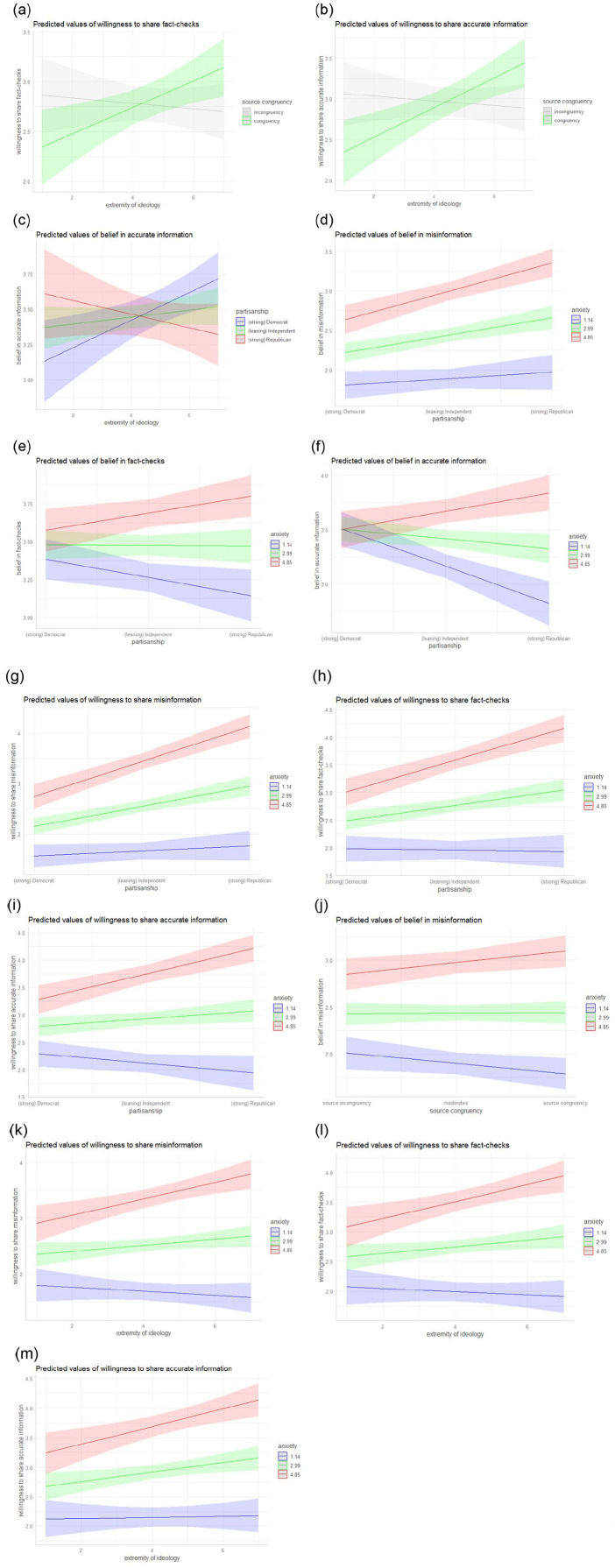
Interactions (for anxiety, mean and 1 standard deviation above and below plotted).^1^ ^1^Please note that the interaction plotted in (a) was not significant in the bootstrapping regression, with *p* = .053.

**Figure 2.**
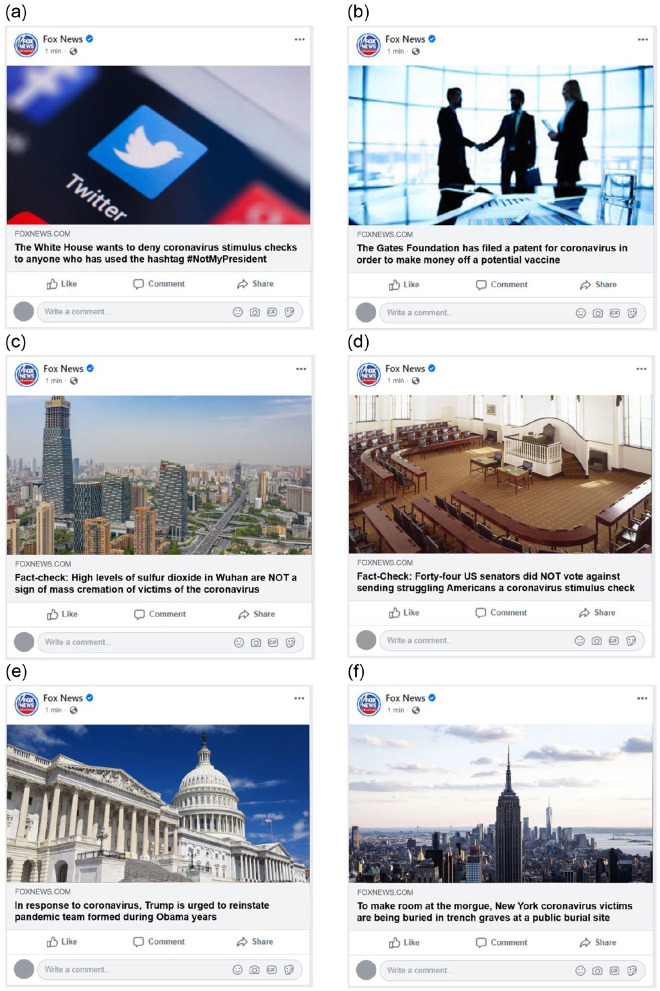
Examples of stimuli with Fox News as the source: (a) misinformation. (b) misinformation. (c) fact-check. (d) fact-check. (e) accurate information. (f) accurate information. Note: We had the same stimuli with MSNBC as the source instead of Fox News, for illustration, we only included Fox News here.

The analyses were conducted in SPSS 26 and the interactions were visualized in R 3.6.3 (Figure 1). We used multiple linear regressions to test our hypotheses (see Table 1 for the regression on belief in misinformation, fact-checks, and accurate information, and Table 2 for the regression on willingness to share misinformation, fact-checks, and accurate information). The residuals of our dependent variables were not normally distributed, so we also ran a bootstrapping regression with 2000 samples for the main effects and each of the six interactions separately for all of our six dependent variables to compare the results. Those 36 bootstrapping regressions showed only very small differences in the betas, which rarely led to a slightly higher or lower significance level. Although the interaction of extremity of ideology and source congruency was significant in the multiple linear regression on willingness to share fact-checks, it was not significant in the respective bootstrapping regression, so we need to be cautious with interpretation of these results. For clarity, we report the multiple linear regressions in more detail below.

**Table 1.** Belief in statements made in stimuli posts as explained by partisanship, extremity of ideology, anxiety, and source congruency.

	Misinformation	Fact-checks	Accurate information
Partisanship and ideology			
Partisanship (Republicans coded high)	.11**	−.05	−.02
Extremity of ideology	.01	−.01	.05
*R*^2^ (in %)	4.50***	.09	.54
Anxiety and source congruency			
Anxiety	.41***	.22***	.20***
Source congruency	−.01	.09*	.02
*R*^2^ change (in %)	17.78***	5.94***	4.94***
Interactions			
Extremity of ideology × source congruency	.04	−.01	.06
Extremity of ideology × partisanship	.06	.07	−.09*
Partisanship × source congruency	.04	.01	−.01
Partisanship × anxiety	.09*	.11**	.19***
Source congruency × anxiety	.09**	.03	.02
Extremity of ideology × anxiety	.06	.02	−.02
*N*	719	719	719

Cell entries are standardized regression coefficients. To minimize collinearity, we report before-entry coefficients for all interactions.

* *p* < .05; **p < .01; ***p < .001.

**Table 2.** Willingness to share stimuli posts as explained by partisanship, extremity of ideology, anxiety, and source congruency.

	Misinformation	Fact-checks	Accurate information
Partisanship and Ideology			
Partisanship (Republicans coded high)	.18***	.12**	.08*
Extremity of ideology	.06	.06	.08*
*R*^2^ (in %)	7.63***	4.71***	3.44***
Anxiety and source congruency			
Anxiety	.46***	.43***	.42***
Source congruency	.03	.00	−.01
*R*^2^ change (in %)	22.74***	19.68***	18.74***
Interactions			
Extremity of ideology × source congruency	.04	.07†	.09**
Extremity of ideology × partisanship	.01	.01	−.01
Partisanship × source congruency	.03	−.01	.00
Partisanship × anxiety	.13***	.15***	.14***
Source congruency × anxiety	.03	−.02	.02
Extremity of ideology × anxiety	.10**	.09**	.07*
*N*	719	719	719

Cell entries are standardized regression coefficients. To minimize collinearity, we report before-entry coefficients for all interactions.

† The interaction is significant on *p* < .05 in the multiple linear regression, but not in the bootstrapping regression with *p* = .053.

* *p* < .05; **p < .01; ***p < .001.

*Source congruency* influenced individuals’ belief in one type of claim, partially supporting H1a, but it had no effect on their willingness to share any claims, contrary to H1b. Specifically, individuals believed more in fact-checks published by an ideologically congruent source than they did in fact-checks from an incongruent source (β = .09, *p* < .05). However, this was not true for belief in misinformation (β = –.01, *p* > .05) or accurate information (β = .02, *p* > .05). None of the estimated effects of source congruency on sharing were significant (misinformation: β = .03, *p* > .05; fact-checks: β = .00, *p* > .05; accurate information: β = –.01, *p* > .05).

*Source congruency and extremity of ideology*^2^ did not interact to influence belief in any type of claim, contrary to H2a (misinformation: β = .04, *p* > .05; fact-checks: β = –.01, *p* > .05; accurate information: β = .06, *p* > .05). However, there was an interaction of source congruency and extremity of ideology on willingness to share some claims, partly supporting H2b. Specifically, extremity of ideology moderated willingness to share accurate information (β = .09, *p* < .01) and possibly fact-checks (β = .07, *p* < .05), but not misinformation (β = .04, *p* > .05). Compared to those with low extremity of ideology (arguably “centrists” or “non-committals”), individuals with high ideological extremity (“extremists”) showed greater willingness to share fact-checks or accurate claims published by an ideologically congruent source (Figure 1(a) and (b), respectively). However, when these same kinds of claims came from an *incongruent* source, ideological centrists or non-committals were more willing than extremists to share them.

*Partisanship* influenced rates of belief only for certain types of information (supporting H3a but not H3b), and it influenced willingness to share misinformation (supporting H5), as well as other kinds of posts. Specifically, the more individuals identified as Republican, the more likely they were to believe misinformation in our stimuli (β = .11, *p* < .01), but this was not true for accurate information (β = –.02, *p* > .05) or fact-checks (β = –.05, *p* > .05). Furthermore, stronger Republicans were indeed more willing to share misinformation (β = .18, *p* < .001), as we expected, but they were also more willing to share fact-checks (β = .12, *p* < .01) and accurate information (β = .08, *p* < .05).

*Extremity of ideology and partisanship* did not interact to influence belief in misinformation (β = .06, *p* > .05), contrary to H4a, but they did interact on belief in accurate information (β = –.09, *p* < .05), supporting H4b. Specifically, ideologically extreme Republicans believed accurate posts less than ideologically extreme Democrats, but ideologically centrist or non-committed Republicans believed accurate posts more than ideologically centrist or non-committed Democrats (Figure (c)).

*Anxiety* positively influenced belief in all kinds of claims, supporting H6. The more anxiety individuals reported, the more they believed in misinformation (β = .41, *p* < .001), fact-checks (β = .22, *p* < .001), and accurate information (β = .20, *p* < .001). Furthermore, answering RQ1, the higher the anxiety, the more willing individuals were to share all kinds of claims (misinformation: β = .46, *p* < .001; fact-checks: β = .43, *p* < .001; accurate information: β = .42, *p* < .001).

In the sections to follow, we answer RQ2a and RQ2b about the combined influence of anxiety and political views (partisanship, source congruency, and extremity of ideology) on belief in and willingness to share various types of claims.

*Partisanship and anxiety* interacted in their influence not only on belief in any given claim (misinformation: β = .09, *p* < .05; fact-checks: β = .11, *p* < .01; accurate information: β = .19, *p* < .001), but also on willingness to share claims (misinformation: β = .13, *p* < .001; fact-checks: β = .15, *p* < .001; accurate information: β = .14, *p* < .001). The relationship between partisanship and belief in claims was significantly stronger among high-anxiety respondents than the rest of the sample, with belief in claims being highest among strong Republicans (Figure 1(d) to (f)). High-anxiety Republicans also showed the greatest willingness to share all types of posts (Figure 1(g) to (i)), as well as the highest levels of belief, especially in misinformation.

*Source congruency and anxiety* interacted for belief in misinformation (β = .09, *p* < .01), but not for belief in fact-checks (β = .03, *p* > .05) or accurate claims (β = .02, *p* > .05), and they did not interact on willingness to share claims (misinformation: β = .03, *p* > .05; fact-checks: β = –.02, *p* > .05; accurate information: β = .02, *p* > .05). Although highly anxious individuals did believe more in misinformation from an ideologically incongruent source than individuals with lower levels of anxiety, they believed *even more* in misinformation when it came from an ideologically congruent source (Figure 1(j)).

*Extremity of ideology and anxiety* did not interact for belief in any claims (misinformation: β = .06, *p* > .05; fact-checks: β = .02, *p* > .05; accurate information: β = –.02, *p* > .05), but they did interact for willingness to share all types of claims (misinformation: β = .10, *p* < .01; fact-checks: β = .09, *p* < .01; accurate information: β = .07, p < .05). Anxiety made a bigger difference for ideological extremists’ willingness to share all claims than it did for ideological centrists or non-committals (Figure 1(k) to (m)).

## Conclusion

The COVID-19 pandemic emerged—at least in the United States—as a perfect exemplar of a policy challenge that (a) required solutions that were heavily dependent on widespread availability of accurate and actionable information, (b) was highly politicized almost from the start, and (c) was surrounded (partly as a result) by significant public uncertainty about many of the factual claims offered in public discourse (Scheufele et al., 2020). This amalgam of factors created many less-than-ideal public health outcomes in the United States and elsewhere. At the same time, the pandemic has provided social scientists with an opportunity to better understand what has begun to emerge as an important set of factors driving the acceptance and spread of (mis)information: emotions, partisanship, and biased processing (often resulting from partisanship). Our study, thus, used the real-world context of an ongoing political and public health crisis as a testing ground for carefully delineating the relative and joint influences of these factors.

Before discussing our results, however, it is important to (re)visit some considerations related to our data and analytic choices. First, it is likely that participants have been ‘pre-treated’—that is, they have seen COVID-19 messages that are similar to our stimuli, or their anxiety is already elevated—which would make it particularly difficult for our stimuli to “move” those who are most likely to form strong beliefs (Druckman and Leeper, 2012). For our purposes, pretreatment would thus make it difficult to detect effects among strong partisans, suggesting that our results might actually be *underestimates*.

Second, when using moderators that were measured observationally rather than experimentally manipulated, there is reason for caution in how we interpret the findings (Kam and Trussler, 2017). Therefore, we advise readers to be careful in the degree to which they interpret the moderation effects we report involving observational variables as causal rather than descriptive. More specifically, there may be a temptation to interpret moderating factors such as partisanship or ideological extremity as having *caused* greater belief in misinformation or sharing of claims, when our experimental design does not fully support that conclusion, instead demonstrating only associations.

Third, our data provide fairly granular insights into how anxiety and partisanship interact with ideological source congruency in shaping belief in and sharing of different types of informational content. It is tempting to go one step further and to speculate, for example, about whether the interaction effect we found between ideological extremity and anxiety is more pronounced for Republicans or Democrats. We opted against reporting such results for the following two reasons: (1) We were unable to distill conclusive and consistent predictions from the literature that would allow us to hypothesize and test such three-way interactions and (2) related to our first point, it is likely that additional tests based on incomplete theoretical reasoning might produce distribution-based statistical results that are misleading (National Academies of Sciences, Engineering, and Medicine 2019).

Fourth, our study was conducted during a pandemic that provided us with an informational environment that gave our (mis)information stimuli—which were partially based on real-world examples—and our anxiety manipulation ecological validity that is often missing from experimental work with more hypothetical or retrospective examples. Having said this, COVID-19 also produced a very unique information environment that might not easily map onto previous political battles over science. RetractionWatch co-founders, Adam Marcus and Ivan Oransky (2020), summarized this nicely during the early stages of the pandemic: “Much of the research that emerges in the coming weeks will turn out to be unreliable, even wrong”.

In this sense, COVID-19 is fundamentally different from vaccine safety or other areas of settled science. We are dealing with highly uncertain and rapidly evolving science that is being used to inform disruptive policy (economic shutdowns, contact tracing, immigration and travel restrictions, etc.). The best available science we have on COVID-19 today will, in many cases, turn out to be wrong. This uncertain climate raises the question as to whether evidence about acceptance and sharing of (mis)information during a crisis like COVID-19 could generalize to other issues, such as debates over campaign finance reform or vaccine safety, where correct information can be more clearly distinguished from false or misleading claims. On the flipside, however, we would argue that understanding citizens’ potential vulnerabilities to misinformation during disruptive health crises, including their relevant motivations, is an important enough problem to be worth studying in its own right.

Our final point relates to the operationalization of ideological source congruency. One could argue that using an average composite of economic and social ideology as the basis of source congruency introduces random error. In particular, two extreme answers on opposite ends of the economic and social ideology question would likely average to a similar value as two mid-scale answers on each of the two scales. In order to address this concern, we tested our models separately for two measures of source congruency, one built from ideology on social issues, and one from ideology on economic issues. We found only minor differences across all models, with most coefficients only changing on the second decimal place and all coefficients pointing in the same direction. We are, therefore, confident that the upsides of building a more reliable index offset the potential downsides of lumping together a small set of respondents, especially given that their aggregation does not substantively change our results.

Somewhat relatedly, some might argue that there is asymmetry in our operationalization of ideological source congruency and, therefore, in our assessment of its effects, given that we chose to use only Fox News (right-leaning) and MSNBC (left-leaning) in our stimuli. Consider, for example, that while 65% of Republicans trust Fox News and 61% of Democrats distrust it, the divide is less intense for MSNBC: 48% of Democrats trust it, while 47% of Republicans distrust it (Jurkowitz et al., 2020). This slight asymmetry speaks to the complexity of operationalizing political source cues in communication research when liberals’ and conservatives’ media-related attitudes vary not only in terms of trust but also in the breadth of sources they use, with poll data showing that strong conservatives are “tightly clustered” around Fox News, while liberals “rely on a greater range of outlets” (Pew Research Center, 2014). The same poll, however, also shows that audiences of Fox News and MSNBC are roughly ideologically equidistant from center. Therefore, even if liberals’ and conservatives’ levels of (dis)trust in these outlets are not perfectly analogous, there is nonetheless some evidence that these outlets represent similar degrees of political “leaning” to the left and right.

With those considerations in mind, this study carefully delineates drivers of partisan motivated reasoning and partisan selective sharing in social media environments, including affective influences. In line with previous research (Calvillo et al., 2020), we find that the more individuals identified as Republican, the more they believed in and shared misinformation—a relationship that is likely due to communications by Grand Old Party (GOP) leaders on COVID-19. However, we also see that situational factors such as anxiety matter for uncertain and risky issues like COVID-19, as they can moderate the effect of more stable factors like partisanship and ideological extremity during individuals’ information encounters.

Our data show that heightened anxiety can help attenuate (partisan) motivated reasoning, which has implications for scholars in communication, political psychology, and other fields who have long explored interventions to minimize confirmation biases and tribal identity protection (for an overview, see Scheufele and Krause, 2019). Indeed, scholars often hope that reducing motivated reasoning will yield normatively desirable democratic outcomes, such as improving the chance that people will critically assess new information or exhibit greater openness to others’ views (Schneiderhan and Khan, 2008). Unfortunately, our results show that while anxiety is indeed linked to greater openness to a diverse set of information, this “openness effect” is potentially a double-edged sword: Highly anxious individuals in our study were more open to both accurate information *and* misinformation, consistent with other recent work (Weeks, 2015).

Our findings indicate that it is crucial to focus not only on ability as a factor in the spread of misinformation, but also on motivational drivers. Many proposed interventions—including “get the facts” labels on tweets or links to fact-checking websites—are based on the assumption that audiences are simply *unable* to distinguish misinformation from reliable news, despite mounting evidence that ability-based approaches are insufficient or can even backfire when they conflict with motivation (Krause et al., 2019). Our study reinforces the idea that believing and sharing information of any kind is strongly related to the motivational influences of partisan priors and emotions. In this way, our results also underscore the utility of evaluating individuals’ encounters with misinformation alongside other kinds of claims. If we had not included fact-checks and accurate claims in this study, we would have missed the important point that anxiety seemed to encourage greater openness to *all* information, not just falsehoods.

The fact that our results are often most pronounced for Republicans could be due to at least three related reasons. First, the former US White House has not only been shown to spread inaccurate or completely false information through official channels, but it has also attacked legacy journalism as one of its banner campaign themes. The tendency we observed among Republicans to share any type of post on social media during a high-anxiety situation (regardless of perceived accuracy) might, therefore, be explained by a partisan-driven desire to counter legacy news narratives. However, one would assume that this tendency would also lead Republicans to share more posts from ideology-consistent sources than Democrats, which we did not see.

Second, it could be argued that Republicans are more likely to share information because they are not as well-informed or tend to be older and less tech savvy than Democrats. This explanation is more speculative and also inconsistent with the fact that, among less anxious respondents, we found few differences between Republicans and Democrats with respect to information belief and sharing. Still, some studies have shown that older respondents tend to share more misinformation on social media (Vosoughi et al., 2018). Although demographic differences are accounted for through random assignment, we ran group comparisons to see if self-identified Republicans and Democrats differed significantly in age or levels of education. They did not.

A third explanation relates to the COVID-19 issue context more specifically. Many official information sources on the coronavirus, such as the Centers for Disease Control (CDC) or the National Institutes of Health (NIH) are part of the executive branch, which, when this study was fielded, was led by President Trump. Also at that time, many CDC messages were cross-branded with the White House (Scheufele et al., 2020). It is, therefore, possible that Republicans were generally more open to messaging than they would be now during a Democrat-led administration, or for issues with less clear threat to their own or their families’ health.

## Notes

1. Detailed coding: (1, 7 = 7), (1.5, 6.5 = 6), (2, 6 = 5), (2.5, 5.5 = 4), (3, 5 = 3), (3.5, 4.5 = 2), and (4 = 1).

2. Although we did not have hypotheses or research questions on this, we note that, as shown in Table 2, extremity of ideology had a significant positive main effect on individuals’ willingness to share accurate information (β = .08, *p* < .05), but not on their willingness to share misinformation (β = .06, *p* > .05) or fact-checks (β = .06, *p* > .05). Extremity of ideology also did not influence belief in any claims. Furthermore, although source congruency and extremity of ideology did interact to influence some forms of sharing (Figure 1(a) and (b)), source congruency and *partisanship* did not interact to affect any outcomes.
